# Factors Influencing Emergency Empathy Towards Patients and Their Relatives: A National Survey Study in Türkiye

**DOI:** 10.3390/healthcare13131559

**Published:** 2025-06-30

**Authors:** Emin Fatih Vişneci, Osman Lütfi Demirci

**Affiliations:** Konya City Hospital Emergency Medicine Department, University of Health Sciences, Konya 42020, Türkiye; osmanlutfi.demirci1@saglik.gov.tr

**Keywords:** empathy, patient, physician, emergency service

## Abstract

**Aim:** The purpose of this study is to identify the factors affecting the empathy that emergency physicians develop toward patients. **Material and Method:** A total of 200 physicians working in the emergency department were included in the study. The Basic Empathy Scale (BES) consists of 20 items, which are divided into two factors: cognitive and affective empathy. The study data were obtained from the surveys. **Results:** All empathy scores were statistically significantly higher in women than in men (*p* values: 0.006, 0.008, and 0.001, respectively). The affective and basic empathy scores of single individuals are higher than those of married individuals (*p* values: 0.032 and 0.034, respectively). The affective and basic empathy scores of individuals without children are higher than those of individuals with children (*p* values: 0.023 and 0.014, respectively). Individuals in medical schools show higher cognitive empathy scores compared to non-medical studying (*p*: 0.004). Individuals who completed special courses (communication, stress management, and empathy) have higher empathy scores compared to those who did not participate (*p* values: 0.002, 0.021, and 0.001, respectively). All empathy scores are similar regardless of the individual’s experience levels, satisfaction with the work environment, the patient group the individuals has more emotional ties with, or the individual’s ability to understand patients in the environment in which they work. The basic empathy scores of individuals working ≤40 h and ≥60 h are similar but less than the basic empathy scores of individuals working 41–60 h. **Conclusions:** Training during or after medical school and better working hours will help to improve the empathy of emergency physicians. Female, single, childless physicians have an advantage regarding empathy in the ED. For married physicians having children, more flexible working environments can increase empathy levels.

## 1. Introduction

Empathy, a multidimensional construct, involves the cognitive and affective ability to perceive, understand, and respond appropriately to the emotions of others [1]. The physician, by the nature of his profession, has a close relationship with his patient. This relationship is not only medical but also includes social elements. Empathy includes the intellectual ability to relate to, care about, and understand the situation and point of view of the client, as well as the ability to communicate this understanding to the client and to act on this understanding in ways that are useful for the treatment of the client. Therefore, the ability to empathize may affect the basis of healthy communication between the doctor and the patient [2,3]. To understand the patient’s perceptions and needs, to empower the patient to cope more effectively, and to solve the patient’s problems, empathy on the part of the clinician is essential in interpersonal communication.

In general, since the conditions that cause the patient to come to the emergency room take place suddenly, at unexpected times, the emergency rooms are unique for which patients and their relatives are unprepared, and where the feeling of helplessness and the expectation of help are at a high level [4].

Emergency departments (EDs), where physicians often encounter patients in high-stress, emotionally charged situations, empathy plays a vital role in communication, treatment adherence, and patient satisfaction.

In emergency medicine, where physicians frequently interact with patients and their relatives under stressful and uncertain circumstances, empathy is especially critical for effective communication and patient-centered care.

Although numerous studies have examined empathy in general healthcare settings, there is limited research focusing on emergency physicians. Furthermore, factors such as patient overload, time constraints, professional burnout, and the chaotic environment of emergency departments in Türkiye may hinder empathetic engagement. These challenges highlight the need for a deeper understanding of empathy within the context of emergency medicine.

In addressing this need, it is crucial to recognize that not all forms of empathy are equally beneficial. In particular, unbalanced forms of empathy, such as excessive affective empathy, can lead to compassion fatigue or emotional exhaustion, which negatively impact physician well-being and patient care. Previous studies [5,6] emphasize the importance of maintaining a balanced empathetic approach to avoid such risks.

The aim of this study is to identify the factors that influence the level of empathy exhibited by emergency physicians. There are few studies on this subject, and data related to emergency services is particularly limited. By identifying these factors, paths for improvement can be sought to establish healthier communication between patients and physicians in the emergency department.

## 2. Material and Methods

### 2.1. Study Design and Participants

This cross-sectional study was conducted between September and December 2024 with 200 emergency medicine physicians practicing in different hospitals across Türkiye. Participants were recruited using a purposive sampling method via online professional networks and national emergency medicine associations. The sample included physicians from diverse geographical regions and institutional levels. Eligibility criteria included active clinical practice in emergency medicine and voluntary participation. A power analysis was conducted prior to the study to ensure a sufficient sample size (power = 0.80, alpha = 0.05).

### 2.2. Data Collection Tools

The data collection tool consisted of three parts: (1) a sociodemographic questionnaire; (2) items exploring professional experience and work conditions; and (3) the Basic Empathy Scale (BES). The Basic Empathy Scale (BES) is an internationally used scale to measure empathy. It is a multidimensional scale, since it clearly distinguishes cognitive from affective empathy. It is one of the most recently developed scales that measures cognitive and affective empathy levels together. The validity and reliability of this scale are determined in many previous studies [7,8]. The BES was developed by Jolliffe and Farrington and later adapted into Turkish by Dinc and Ayhan (2019). The scale includes 20 items measuring cognitive and emotional empathy. Example items include: “I can understand how others feel even when they don’t tell me” (cognitive empathy) and “When someone is upset, I feel upset too” (emotional empathy). Responses were rated on a 5-point Likert scale (1 = strongly disagree, 5 = strongly agree).

In addition to the BES, the following questions were directed to the participants in the survey:

- What is the general level of empathy (cognitive and emotional) among emergency service physicians in Turkey?
- Which demographic and professional factors are associated with higher or lower empathy scores?
- Do gender, parenting, and workload have different effects on emotional empathy and cognitive empathy?

### 2.3. Ethical Considerations

Ethical approval was obtained from the University of Health Sciences, Konya City Hospital Local Ethics Committee (Approval No: 04-50, dated 7 March 2024). All participants provided informed consent prior to participation. The study complied with the Declaration of Helsinki.

### 2.4. Statistical Analysis

To analyze the obtained data, the applicability of parametric or non-parametric tests was decided by looking at the suitability of the values to normal distribution and equality of variance. The suitability of the data for normal distribution was tested using the Kolmogorov–Smirnov test and again using the Levene test for homogeneity of variance. To apply parametric tests, both compliance with normal distribution and homogeneity of variance are required at the same time. Descriptive statistics were presented as mean ± standard deviation (SD), minimum–maximum values, and percentages.

To analyze the data, Student’s t-test was used for two independent groups; statistical significance was set at *p* < 0.05. The effect size was reported as Cohen’s d for t-tests and eta-squared for ANOVA, where applicable. Additionally, the Pearson correlation coefficient was performed for relationship analysis. AMOS 22 and SPSS 22 package programs were used in the statistical tests of the study.

## 3. Results

### 3.1. Descriptive Statistics

A total of 200 (78 female and 122 male) physicians, all graduates from the medical school, were included in the study. Among those participants, 25 were academicians in ED, 74 were specialists, 82 were residents, and 19 were general practitioners working in ED. The descriptive data of the study participants are summarized in Table 1.

### 3.2. Validity and Reliability Analysis of Scales

A multi-factor confirmatory factor analysis was conducted with the SPSS AMOS 22 program to determine the construct validity of the previously developed scale. When the fit values produced by the measurement models created to test the validity of the scales are not within acceptable limits, the changes are made to the scale as a result of the modifications. After the modifications, Questions 6 and 19 from the Cognitive Empathy scale and Question 8 from the Affective Empathy Scale were removed from the Basic Empathy Scale subscale.

To assess the adequacy of the sample size, the Kaiser–Meyer–Olkin (KMO) statistic was computed. The inter-item correlations were examined using Bartlett’s test of sphericity. The KMO value of 0.960 exceeded the acceptable threshold of 0.50, and the Bartlett’s test yielded a significance level of *p* = 0.001, indicating that the data were suitable for factor analysis.

To evaluate the scale’s applicability, its internal consistency was assessed via Cronbach’s alpha coefficient. The overall reliability coefficient of the scale was calculated as 0.719, suggesting a high level of internal consistency. These findings confirm that the developed scale is bidimensional and possesses strong validity and reliability.

### 3.3. Analyses

Cognitive, affective, and basic empathy scores were all found to be statistically significantly higher in women than in men (*p* values: 0.006, 0.008, and 0.001, respectively). The affective and basic empathy scores of single individuals are higher than those of married individuals (*p* values: 0.032 and 0.034, respectively). The affective and basic empathy scores of individuals without children are higher than that of individuals with children (*p* values: 0.023 and 0.014, respectively).

The cognitive and basic empathy scores of individuals whose self or family situation was changed in the last year are higher than those of individuals without a change in the last year (*p* values: 0.024 and 0.043, respectively). The cognitive empathy scores of individuals who had communication lessons in medical school are higher than the cognitive empathy scores of individuals who are not studying medicine (*p*: 0.004). The cognitive, affective, or basic empathy scores of individuals who took the courses (e.g., communication, stress management, and empathy) are higher than that of individuals who did not take the courses (*p* values: 0.002, 0.021, and 0.001, respectively) (Table 2).

Cognitive, affective, or basic empathy scores are similar regardless of individuals’ experience levels, individuals’ satisfaction with the work environment, the patient group individuals have more emotional ties with, or the individual’s ability to understand patients in the environment in which they work (Table 3). Individuals’ cognitive or affective empathy scores are similar regardless of their weekly working hours. As a result of the pair-wise comparison, the basic empathy scores of individuals working 40 h or less and 60 h or more are similar but less than the basic empathy scores of **individuals working 41–60 h.**

## 4. Discussion

In this study, we aimed to identify the factors affecting the empathy that emergency department physicians develop towards patients and their relatives and we determined that female gender, being single, being childless, any change in the self or family situation in the last year, taking communication skills lesson in medical faculty, and taking courses (e.g., communication, stress management, and empathy) were providing more of an advantage for the empathy scores to the ED physicians. The best basic empathy scores were obtained among the healthcare professionals working 41–60 h.

In recent years, the ability to empathize has been identified as being particularly important for people working in the healthcare sector, because working in hospitals requires constant interaction with patients and/or their relatives. In a recent study, it was reported that there was a positive association between ED provider self-reported empathy and after-care instant patient-to-provider satisfaction, emphasizing that higher empathy scores were associated with higher patient satisfaction [9]. Similarly, patient satisfaction and clinical outcomes are associated with higher levels of physician empathy [10]. In this aspect, we tried to determine the factors affecting the empathy levels of ED physicians.

We determined that the female is associated with higher cognitive, affective, and basic empathy scores. In general, the results of the previous literature were also in parallel with this finding. Katsari et al. reported that being female, being married, and the duration of employment were reported as important predictors of increased physician empathy [11]. In another study performed in Korea on 229 physicians with the Jefferson Scale of Physician Empathy, women scored significantly higher than men [12]. In a cross-sectional study, conducted with 143 physicians working in the ED, it was found that female doctors have more empathy than male doctors, and married people have more empathy than singles [13]. In another study performed with a revised version of the Jefferson Scale of Physician Empathy, which was mailed to 1007 physicians, women scored higher than men to a nearly significant degree [1]. In contrast, Subramaniam et al. did not determine any significant difference in empathy between the two genders in their cross-sectional study of 127 medical doctors in a university hospital [14]. The gender difference in the level of empathy was explained by the cultural expectations about gender roles, by the evolutionary primary role of women as caretakers of children and the elderly, or by gender differences in the brain networks supporting empathy on the neurobiological level. In our study, female physicians in Türkiye demonstrated higher levels of empathy compared to their male counterparts. This finding may be explained by culturally rooted gender norms that encourage emotional sensitivity, nurturance, and interpersonal attentiveness among women. Previous Turkish studies [15,16] have similarly reported that sociocultural expectations and traditional roles contribute to higher emotional engagement among female healthcare professionals. These results suggest that cultural context plays a significant role in shaping empathic tendencies and should be considered when designing training and support systems for emergency physicians. All these factors may play a role in the higher empathy ability of female physicians compared with male colleagues [17,18,19].

Interestingly, we determined that the empathy scores of single and/or childless physicians were higher than those of married physicians or physicians having children. This result contradicts previous findings [11,13]. In a recent review, having children and being married were found to have a positive impact on empathy among family medicine physicians [20]. Park et al. also reported that being female, married, and having children were factors related to higher empathy among medical residents in a multicenter study [21]. This difference may be because the emergency department is an extremely busy and active department due to its dynamics, and married people with children are very tired at home and do not have the energy to provide sufficient empathy in the emergency department. However, larger studies are warranted on this subject.

Sung et al. indicated significant correlations between attachment to dogs, human empathy, and quality of life in a survey-based study performed on 263 dog owners [22]. However, we did not determine any association between empathy scores and having a pet. This may be due to the low number of physicians having pets in our study (less than 30%). Larger studies may reveal different results.

We did not determine a significant effect of the level of experience on the empathy scores of ED physicians. Similarly, Walocha et al. studied 92 physicians and reported that there was no correlation between the number of years of experience working as a doctor and the level of empathy [23]. However, in a cross-sectional study by Obimakinde et al. on 188 physicians, duration of practice and work hours respectively correlated positively and negatively with empathy scores [24]. Bayne et al. also found that the more experienced the doctor, the more empathetic the doctor tended to behave [25]. The majority of physicians (more than 75%) who participated in our study had a working period of less than 15 years. This may be the reason that no significant difference has been detected in this study regarding the work experience and empathy level.

The medical doctors in the study who had a negative medical condition in themselves or their families in the last year had significantly higher empathy scores. Among medical doctors, the personal story of their illness can evoke empathy [26]. It is recognized that there is a strong link between developing empathy and daily experiences [27]. However, in contrast with our results, Akgün et al. did not determine any significant relationship between the presence of any negative events in the last year and empathy scores among medical school students [28]. However, in daily practice, medical doctors believe that daily experiences that take place also outside of work may play an important role in empathy level.

The empathy scores of physicians who had communication lessons in medical school or who took the courses (e.g., communication, stress management, and empathy) are higher in our study. In a recent study performed on 241 final-year medical students, with two self-reported empathy questionnaires, it was reported that multi-year reflective learning interventions during clinical training nurture empathy in medical students [29]. In another study performed in our country on 257 students from a medical and a nursing school, a 10 h empathy and communication training program delivered over five consecutive weeks led to a statistically significant improvement in participants’ performance, as measured by both the Empathic Communication Skills Scale and the Empathic Tendency Scale. The study’s findings also suggest that well-structured educational interventions can effectively enhance empathy-related competencies and tendencies among medical and nursing students of both sexes [30]. In light of these data, we believe that education, during or after medical school education, may improve empathy in physicians.

An interesting finding of this work was that any verbal/physical attack by patients or relatives or complaints from patients or relatives did not significantly affect the empathy levels of the physicians. It has been suggested that effective interpersonal empathic communication may play a significant role in the reduction of violence in the ED [31]. Yu et al. reported that negative interactions with patients inhibited empathy in healthcare professionals [32]. In this sense, our findings are promising, because even if physicians are subjected to violence, there is no significant decrease in their empathy levels, which increases their chances of coping if they encounter this situation again.

The empathy scores of physicians were similar regardless of their satisfaction with the work environment. In a recent study, interestingly, empathy was reported to be significantly and negatively correlated with job satisfaction [33].

The basic empathy scores of individuals working 40 h or less and 60 h or more were similar but less than the basic empathy scores of individuals working 41–60 h. In a cross-sectional study performed on 282 residents, residents working less than 80 h per week had higher empathy scores as compared to residents working more than 80 h, and this result was statistically significant [34]. In parallel with the previous literature, elongated work hours negatively affected empathy scores [24,35].

This is a multicenter study with some limitations. First of all, we analyzed the physician self-assessed empathy with the surveys; however, patient-perceived physician empathy should also be analyzed to determine the effects of this self-assessed empathy of physicians, because self-assessed empathy levels and patients’ perceptions may not be similar every time [36]. The potential for participants to be biased in responding and the convenient sampling method used for this study can be seen as another limitation.

## 5. Conclusions

The emergency department is a very busy and stressful environment, and emergency physicians work under a great deal of pressure. Training during or after medical school and better working hours will help to improve the empathy of emergency physicians. Female, single, childless physicians have an advantage regarding empathy in ED. For married physicians who have children, more flexible working environments can increase empathy levels.

## Figures and Tables

**Table 1:** Descriptive data of study participants.

General Features	n	%
**Gender (F/M)**	**78/122**	**39/61**
**Years in the profession**		
1–5	81	40.5
5–10	36	18
10–15	42	21
15–20	23	11.5
>20	18	9
**Marital status (Married/Single)**	122/78	61/39
**Do you have children? (Y/N)**	99/101	49.5/50.5
**Do you have a pet? (Y/N)**	56/144	28/72
**Participation in social activities (Y/N)**	156/44	78/22
**Have there been any negative events about yourself or your family in the last year? (Y/N)**	75/125	37.5/62.5
**Did you take communication lessons in the Faculty of Medicine (Y/N)**	166/34	83/17
**Have you taken courses after graduation (e.g., communication, stress management, and/or empathy)? (Y/N)**	128/72	64/36
**Weekly working time (hours)**		
≤40	21	10.5
41–60	134	67
≥60	45	22.5
**Satisfaction with your work environment**		
Yes	64	32
No	33	16.5
Partially	103	51.5
**Which patient group do you have a more emotional connection with?**		
Women	7	3.5
Children	84	42
Elderly	43	21.5
All	66	33
**Understanding patients in your work environment**		
Yes	129	64.5
No	10	5
Partially	61	30.5
**Have you been subjected to verbal/physical attacks by patients/relatives in your professional life? (Y/N)**	187/13	93.5/6.5
**Did you receive complaints from patients/relatives throughout your career? (Y/N)**	127/73	63.5/36.5

**Table 2:** Comparison of BES scores regarding descriptive features of study participants.

Factors	Cognitive Empathy	Affective Empathy	Basic Empathy Score
**Gender**			
**Female/Male**	23.96 ± 0.28/22.80 ± 0.30	28.53 ± 0.40/26.86 ± 0.40	52.50 ± 0.54/49.67 ± 0.58
***p***	**0.006**	**0.008**	**0.001**
**Marital status**			
**Married/Single**	23.13 ± 0.29/23.44 ± 0.51	27.01 ± 0.39/28.32 ± 0.45	50.14 ± 0.56/51.77 ± 0.64
***p***	0.511	**0.032**	**0.034**
**Presence of children**			
**Y/N**	22.97 ± 0.34/23.53 ± 0.27	26.83 ± 0.45/28.20 ± 0.37	49.80 ± 0.65/51.73 ± 0.54
***p***	0.286	**0.023**	**0.014**
**Presence of a pet**			
**Y/N**	23.62 ± 0.36/23.11 ± 0.27	27.77 ± 0.63/27.42 ± 0.33	51.39 ± 0.81/50.53 ± 0.50
***p***	0.532	0.989	0.652
**Participation in social activities**			
**Y/N**	23.30 ± 0.25/23.09 ± 0.45	27.73 ± 0.35/26.77 ± 0.56	51.03 ± 0.49/49.86 ± 0.84
***p***	0.457	0.281	0.304
**A negative condition in the family in the last year**			
**Y/N**	23.91 ± 0.31/22.08 ± 0.29	27.97 ± 0.48/27.24 ± 0.38	51.88 ± 0.64/50.11 ± 0.55
***p***	**0.024**	0.321	**0.043**
**Communication lessons**			
**Y/N**	23.58 ± 0.21/21.65 ± 0.73	27.57 ± 0.31/27.23 ± 0.92	51.16 ± 0.43/48.88 ± 1.33
***p***	**0.004**	0.952	0.147
**Courses**			
**Y/N**	23.81 ± 0.23/22.28 ± 0.42	28.04 ± 0.35/26.60 ± 0.53	51.84 ± 0.46/48.87 ± 0.80
***p***	**0.002**	**0.021**	**0.001**
**Verbal/physical attack by patients or relatives**			
**Y/N**	23.20 ± 0.23/24.00 ± 0.42	26.64 ± 0.31/25.77 ± 1.13	50.84 ± 0.45/49.77 ± 1.11
***p***	0.497	0.102	0.267
**Complaints from patients or relatives**			
**Y/N**	23.40 ± 0.25/23.06 ± 0.41	27.20 ± 0.36/26.34 ± 0.49	50.09 ± 0.47/49.94 ± 0.79
***p***	0.858	0.108	0.156

**Table 3:** Comparison of BES scores regarding workplace experiences of study participants.

Factors	Cognitive Empathy	Affective Empathy	Basic Empathy
**Experience (years)**			
1–5	23.56 ± 0.32	27.89 ± 0.44	51.45 ± 0.61
5–10	23.30 ± 0.36	27.28 ± 0.55	49.67 ± 0.58
10–15	22.69 ± 0.55	27.38 ± 0.78	52.50 ± 0.54
15–20	23.04 ± 0.70	27.48 ± 1.03	49.67 ± 0.58
>20	23.33 ± 0.95	26.72 ± 0.98	52.50 ± 0.54
*p*	0.728	0.824	0.741
**Weekly working hours**			
<40	23.24 ± 0.67	26.05 ± 0.85	49.28 ± 1.19
40–60	23.66 ± 0.23	27.88 ± 0.32	51.54 ± 0.44
>60	22.07 ± 0.56	27.13 ± 0.79	49.20 ± 1.21
*p*	0.059	0.169	0.048
**Satisfaction with your work environment**			
Yes	23.42 ± 0.38	27.75 ± 0.51	51.17 ± 0.72
No	23.30 ± 0.48	27.66 ± 0.83	50.97 ± 1.11
Partially	23.13 ± 0.32	27.33 ± 0.41	50.46 ± 0.60
*p*	0.812	0.847	0.609
**Which patient group do you have a more emotional connection with?**			
Women	23.57 ± 0.92	29.14 ± 1.14	52.71 ± 1.12
Children	23.41 ± 0.36	27.86 ± 0.49	51.28 ± 0.72
Elderly	22.70 ± 0.44	27.74 ± 0.66	50.44 ± 0.88
All	23.38 ± 0.36	26.75 ± 0.46	50.14 ± 0.67
*p*	0.355	0.267	0.567
**Understanding patients in your work environment**			
Yes	23.45 ± 0.29	27.14 ± 0.39	50.58 ± 0.56
No	22.70 ± 0.73	29.40 ± 1.54	52.10 ± 1.87
Partially	22.93 ± 0.35	28.03 ± 0.46	50.96 ± 0.67
*p*	0.170	0.145	0.767

## Data Availability

Data is contained within the article.
